# Antioxidant Gene Signature Impacts the Immune Infiltration and Predicts the Prognosis of Kidney Renal Clear Cell Carcinoma

**DOI:** 10.3389/fgene.2021.721252

**Published:** 2021-08-19

**Authors:** Xueting Ren, Li Ma, Nan Wang, Ruina Zhou, Jianhua Wu, Xin Xie, Hao Zhang, Di Liu, Xiaobin Ma, Chengxue Dang, Huafeng Kang, Zhangjian Zhou

**Affiliations:** ^1^Department of Oncology, The Second Affiliated Hospital of Xi’an Jiaotong University, Xi’an, China; ^2^Department of Surgical Oncology, The First Affiliated Hospital of Xi’an Jiaotong University, Xi’an, China

**Keywords:** kidney renal clear cell carcinoma, cellular antioxidant mechanisms, immune infiltration, nomogram, prognosis

## Abstract

**Background:** Oxidative stress is related to oncogenic transformation in kidney renal clear cell carcinoma (KIRC). We intended to identify a prognostic antioxidant gene signature and investigate its relationship with immune infiltration in KIRC.

**Methods:** With the support of The Cancer Genome Atlas (TCGA) database, we researched the gene expression and clinical data of KIRC patients. Antioxidant related genes with significant differences in expression between KIRC and normal samples were then identified. Through univariate and multivariate Cox analysis, a prognostic gene model was established and all patients were divided into high- and low-risk subgroups. Single sample gene set enrichment analysis was adopted to analyze the immune infiltration, HLA expression, and immune checkpoint genes in different risk groups. Finally, the prognostic nomogram model was established and evaluated.

**Results:** We identified six antioxidant genes significantly correlated with the outcome of KIRC patients as independent predictors, namely DPEP1 (HR = 0.97, *P* < 0.05), GSTM3 (HR = 0.97, *P* < 0.05), IYD (HR = 0.33, *P* < 0.05), KDM3B (HR = 0.96, *P* < 0.05), PRDX2 (HR = 0.99, *P* < 0.05), and PRXL2A (HR = 0.96, *P* < 0.05). The high- and low-risk subgroups of KIRC patients were grouped according to the six-gene signature. Patients with higher risk scores had poorer prognosis, more advanced grade and stage, and more abundance of M0 macrophages, regulatory T cells, and follicular helper T cells. There were statistically significant differences in HLA and checkpoint gene expression between the two risk subgroups. The performance of the nomogram was favorable (concordance index = 0.766) and reliably predicted the 3-year (AUC = 0.792) and 5-year (AUC = 0.766) survival of patients with KIRC.

**Conclusion:** The novel six antioxidant related gene signature could effectively forecast the prognosis of patients with KIRC, supply insights into the interaction between cellular antioxidant mechanisms and cancer, and is an innovative tool for selecting potential patients and targets for immunotherapy.

## Introduction

Renal cell carcinoma (RCC) is a common cancer of urinary system, accounting for around 4% of all newly diagnosed cancers worldwide (Siegel and Miller, 2021). There will be approximately 76,080 new cases and 13,780 deaths in the United States alone by 2021 (Siegel and Miller, 2021). Kidney renal clear cell carcinoma (KIRC) occupies 70–80% of RCC and is the eighth most common cancer type (Zhao et al., 2018). Asymptomatic patients are found more likely to have progressed to advanced stages which causes high mortality and recurrence rates of RCC. This reasonably indicates the importance of early diagnosis and prognosis evaluation of RCC (Sejima et al., 2013; Zhao et al., 2018). While, as one of the major burdens of global health, KIRC still lacks effective prognostic biomarkers (Barata and Rini, 2017). Hence, it is prospective to find novel biomarkers for the identification of patients at high risk of worse outcome and construct a risk model to assess their prognosis for reasonable clinical decision and management in KIRC.

Recent studies have confirmed that reactive oxygen species (ROS) and antioxidants participate in the occurrence and progress of cancers and other diseases (Dalle-Donne et al., 2006; Agarwal et al., 2012; Tangvarasittichai, 2015). ROS plays a crucial role in normal cellular signaling pathways, but excessive ROS can injure genomic and mitochondrial DNA, leading to oxidative damage, molecular mutations, and changes in signaling pathways (Alpay et al., 2015), which promotes tumorigenesis (Ishikawa et al., 2008; Kumar et al., 2008). Some studies have shown that certain antioxidants play an essential role in tumor prevention by preventing DNA damage caused by excessive ROS (Snezhkina, 2019; Harris and DeNicola, 2020), while other studies claimed that antioxidants promote tumorigenesis by protecting cancer cell from excessive ROS caused death (Athreya and Xavier, 2017). For example, recent studies have shown that the role of peroxidase 1 (PRDX1) as an antioxidant in breast cancer has two sides. For one thing, PRDX1 may prevent oxidative stress-mediated ERα loss through antioxidant function, thereby contributing to maintaining the ER-positive phenotype of breast tumors and improving the prognosis of breast cancer (O’Leary et al., 2014). For another, some studies have shown that PRDX1 is overexpressed in breast tumors compared with normal tissues, predicting poor prognosis (Cha et al., 2009; Wang G. et al., 2019). Similarly, compared with normal tissues, another typical 2-Cys antioxidant enzyme, peroxiredoxin 2 (PRDX2), is downregulated in melanoma tissues and upregulated in the colon, cervical, lung, and other malignancies (Furuta et al., 2006; Lomnytska et al., 2011; Stresing et al., 2013; Xu et al., 2017). In summary, the role of antioxidants in cancer is controversial and worthy of further study. Accordingly, in this study, the expression pattern of antioxidant genes in KIRC was investigated based on the transcriptome profile of the Cancer Genome Atlas (TCGA) database to reveal the role or mechanism of antioxidant genes in KIRC. Furthermore, antioxidant genes may also be potential biomarkers, which can provide valuable prognostic information for KIRC.

In recent years, immunotherapy has become an effective means of cancer treatment. Due to the poor sensitivity to conventional radiotherapy and chemotherapy, treatment of KIRC has gradually shift from non-specific immunological approaches to targeted therapies, and now to new immunotherapeutic agents (Barata and Rini, 2017; Fan et al., 2021). Cytokine-based immunotherapies, such as IFN-α and IL-2, are effective in a small proportion of patients with metastatic RCC (Fyfe et al., 1995). Immunotherapy suppresses immune tolerance by inhibiting the interaction between immune and tumor cells (Fyfe et al., 1995). Certain immune checkpoint pathways are activated as the main mechanism of immune resistance of cancer. Therefore, blocking immune checkpoints is expected to be a new way of cancer treatment (Pardoll, 2012). Many immune checkpoints are initiated by ligand-receptor interactions, and the receptors involved in such interactions include cytotoxic T lymphocyte-associated protein-4 (CTLA4), the PD1 receptor expressed in activated T cells, and the PDL1 and PDL2 receptors expressed on immune and tumor cells (Pardoll, 2012; Batlevi et al., 2016). Nivolumab, a PD1 checkpoint inhibitor with anti-tumor activity, has been shown to benefit the survival of patients with advanced KIRC who received anti-angiogenic therapy in the past (Motzer et al., 2015). Besides, the efficacy of ipilimumab, a CTLA4 checkpoint inhibitor, and atezolizumab, a PDL1 checkpoint inhibitor, was investigated and promising in KIRC (Hammers et al., 2017; Atkins and Tannir, 2018; Motzer et al., 2018; Pal et al., 2020). Exploring new checkpoint inhibitors is essential to provide additional therapeutic targets for KIRC immunotherapy. Currently, the selection of target patients with high response to specific drugs is still a major challenge for immunotherapy. It is necessary to find new and effective biomarkers to guide the selection of specific patients. Both immune cells and tumor cells are affected in a tumor microenvironment where ROS levels remain high. Some researchers have proved that the anti-cancer effects of immune cells are related to their antioxidant capacity (Yang et al., 2013; Yarosz and Chang, 2018). When the ROS level increases to inhibit the anti-tumor ability of immune cells, immune suppression occurs in the tumor microenvironment (Wang et al., 2021). Considering the association between antioxidants and immune infiltration, the establishment of an antioxidant gene signature can not only assess the prognosis of patients with KIRC, but also select the appropriate immunotherapy population according to the immune infiltration status of different subgroups.

This study determined a novel antioxidant gene signature correlated with the prognosis of patients with KIRC by analyzing the data from TCGA database. Then we investigated the association between this gene signature and immune infiltration. In addition, combined with the gene signature and other important clinical manifestation, a nomogram was built to effectively foresee the prognosis of patients with KIRC.

## Materials and Methods

### Collection of Data

The clinical information and gene expression data of KIRC patients were collected from TCGA database.^1^ Gene expression files were presented as FPKM formatted RNA-seq data. FPKM is defined as the fragments per kilobase of transcript per million mapped reads, which indicates that its calculation normalizes read count by dividing it by the gene length and the total number of reads mapped to protein-coding genes. In this study, we included 539 KIRC samples and 72 normal samples. Moreover, the following demographic and clinical information of all cases were collected: age, sex, follow-up time, survival status, and tumor grade and stage. The analysis process used in this study was displayed in the flowchart (Supplementary Figure 1).

### Identification of Differentially Expressed Antioxidant Genes

We selected four antioxidant gene sets (antioxidant activity, GO antioxidant activity, GO glutathione catabolic process, and GO glutathione metabolic process) from the molecular signatures database for gene set enrichment analysis.^2^ These gene sets are composed of genes that participate in antioxidant-related pathways or biological processes. Supplementary Table 1 shows the complete list target antioxidant genes. Then, in the R language environment, we screened the antioxidant gene expression data from the TCGA database and used the “limma” R package to distinguish differentially expressed genes in patients with KIRC (Ritchie et al., 2015). The cut-off criteria were set as *P* < 0.05, and | logFC (fold change) | > 0.

### Establishment and Validation of the Gene Signature

Univariate Cox regression analysis was used to determine the genes significantly associated with the overall survival of patients with KIRC. Subsequently, multivariate Cox regression analysis was performed to further confirm the independent prognostic genes. The risk score of each KIRC patient was obtained according to the expression level of each prognostic gene and the corresponding coefficient in the multivariate Cox regression analysis. Its calculation is shown in the following equation: risk score = h (t, X) = h_0_(t) × exp (expression of gene 1 × β1 + expression of gene 2 × β2 + ⋯ + expression of gene n × βn). In the formula, X represents the expression of genes and h_0_(t) is a constant during the multivariate Cox regression analysis. n and β_*n*_, respectively, represent the number of independent prognostic genes and the regression coefficient value. Exp () represents an exponential function with e as the base. Among them, With the median risk score as the cutoff, we separated KIRC patients into high- and low-risk subgroups and studied the correlation between these two risk subgroups and clinicopathological features. By using the “Survminer” R package, we performed the Kaplan-Meier survival analysis and log-rank test to assess the survival difference between high- and low-risk subgroups. Meanwhile, ROC analysis was performed with the “survivalROC” package to further evaluate the accuracy of the prognostic model. Then, the overall survival was compared among age, sex, stage, and grade. Finally, the overall survival between high- and low-risk subgroups was studied in the stratified subgroup to further investigate the predictive ability of the gene signature.

### Analysis of the Relationship Between Risk Score and Immune Infiltration

Taking the median risk score of six-gene signature as the cutoff, KIRC patients were separated into high- and low-risk subgroups. Then, the “estimate” software package and unsupervised consensus cluster analysis were used to investigate the tumor microenvironment of the two risk groups. Parameters including the stromal scores, immune scores, estimate scores, and tumor purity were analyzed and studied (Yoshihara et al., 2013). Based on CIBERSORT algorithm, 22 immune cells included in the gene sets of myeloid cells, B cells, T cells and NK cells were studied to assess the immune infiltration of the high- and low-risk subgroups (Newman et al., 2015). Then, single sample gene set enrichment analysis (ssGSEA) containing the “GSVA” R software package was applied by following research (Barbie et al., 2009). Further studies were carried out on the relationship between human leukocyte antigen (HLA) gene expression, immune cells, related pathway types, immune checkpoint genes and risk scores.

### Establishment and Assessment of the Nomogram

In order to evaluate the survival probability of KIRC patients, the nomogram model was constructed by using “rms” package in R language environment combined with clinicopathological features and six-gene signature. After Cox regression analysis, all variables were given a certain score, and then the total score was added to the nomogram to predict 3- and 5-years survival rates. The higher the total score, the worse the prognosis. The efficiency of the nomogram was evaluated using the generated calibration diagrams, the area under the ROC curve (AUC), and C-index. The C-index is positively correlated with the accuracy of the nomogram, and the ideal calibration chart should be close to the 45-degree dotted line. These activities were resampled using bootstrapping.

### Statistical Analysis

This study analyzed the data and generated charts based on R software (version 4.0.2) and Excel software (Microsoft Corporation, California). Flexible statistical methods were used for the statistical analysis. When *P*-value was less than 0.05, the difference had a statistical significance.

## Results

### Features of Patients With KIRC Enrolled in This Study

In the TCGA database, we extracted the transcription and clinical data of 539 KIRC and 72 normal samples and then matched the data with the sample ID for subsequent analysis. In our study, a total of 530 matched patients were enrolled, and their clinical data were collected, including survival status, grade of tumor, American Joint Committee on Cancer (AJCC) stage, classification of tumor, lymph node, and metastasis. Table 1 presents the detailed clinical pathological parameters included in this study.

**TABLE 1:** Clinicopathological parameters of KIRC patients in this study.

Clinical pathological parameters	N	%
**Age (years)**		
≤ 65	348	65.66
> 65	182	34.34
**Gender**		
Male	344	64.91
Female	186	35.09
**Grade**		
G1-2	241	46.17
G3-4	281	53.83
**T classification**		
T1-T2	340	64.15
T3-T4	190	35.85
**N classification**		
N0	239	93.73
N1-3	16	6.27
**M classification**		
M0	420	84.34
M1	78	15.66
**UICC stage**		
I-II stage	322	61.1
III-IV stage	205	38.9
**Survival status**		
Alive	364	68.68
Dead	166	31.32

*KIRC, kidney renal clear cell carcinoma; UICC, Union for International Cancer Control; T, tumor; N, node; M, metastasis.*

### Differentially Expressed Antioxidant Genes in KIRC and Normal Tissues

Based on four antioxidant gene sets (antioxidant activity, GO antioxidant activity, GO glutathione catabolic process, and GO glutathione metabolic process gene sets), we estimated the gene expression levels of all samples in the TCGA database. The findings displayed that there were 92 differentially expressed antioxidant genes in KIRC tissues compared with normal controls, of which 57 were down-regulated and 35 were up-regulated (Supplementary Table 2). Details of fold-change and *p*-value for differentially expressed genes are shown in Supplementary Table 3.

### Determination of the Antioxidant Genes Correlated With the Survival of Patients With KIRC

**FIGURE 1:**
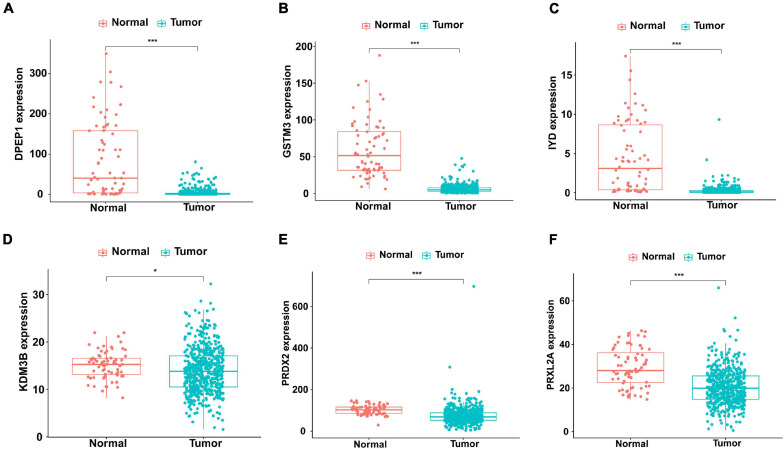
Different expression of the six genes in KIRC (*n=539*) and normal (*n=72*) samples with unpaired *t*-test. The solid line represents the median expression of independent prognostic antioxidant genes. The expression of the six genes was all down-regulated in KIRC patients. **(A)** DPEP1, **(B)** GSTM3, **(C)** IYD, **(D)** KDM3B, **(E)** PRDX2, and **(F)** PRXL2A (*P < 0.05 and ***P < 0.001).

The first step was to determine the antioxidant genes remarkably correlated with the survival of patients with KIRC by univariate Cox regression analysis. We obtained 13 statistically significant genes (APOM, CAT, DPEP1, GLO1, GSTM3, IYD, KDM3B, NFE2L2, PRDX2, PRDX3, PRXL2A, S100A9, and UBIAD1). Except for S100A9, other genes were down-regulated in tumor tissues (Table 2). Next, using multivariate Cox regression analysis, we further probed genes with independent prognostic value from these 13 genes. Finally, it was determined that DPEP1 (HR = 0.97, *P* < 0.05), GSTM3 (HR = 0.97, *P* < 0.05), IYD (HR = 0.33, *P* < 0.05), KDM3B (HR = 0.96, *P* < 0.05), PRDX2 (HR = 0.99, *P* < 0.05), and PRXL2A (HR = 0.96, *P* < 0.05) could independently predict the prognosis of KIRC. The unpaired *t*-test was used to analyze the expression differences of six core genes in 539 KIRC patients and 72 normal samples. The findings suggested that compared with normal tissues, the expression of DPEP1, GSTM3, IYD, KDM3B, PRDX2, and PRXL2A were all downregulated in KIRC patients (Figure 1).

**TABLE 2:** The antioxidant genes correlated with prognosis of KIRC in univariate cox analysis.

Gene	HR	Lower 95%CI	Upper 95%CI	Cox *P*-value
PRDX3	0.984	0.973	0.994	0.002
KDM3B	0.924	0.893	0.956	< 0.001
IYD	0.188	0.082	0.430	< 0.001
CAT	0.981	0.974	0.989	< 0.001
DPEP1	0.951	0.918	0.985	0.005
GSTM3	0.959	0.922	0.996	0.033
PRXL2A	0.949	0.929	0.970	< 0.001
GLO1	0.988	0.976	0.100	0.046
S100A9	1.002	1.000	1.004	0.043
PRDX2	0.990	0.984	0.995	< 0.001
NFE2L2	0.974	0.954	0.995	0.015
APOM	0.989	0.978	0.999	0.028
UBIAD1	0.808	0.696	0.939	0.005

*HR, hazard ratio; CI, confidence interval.*

### Establishment of an Antioxidant Gene Signature as a Risk Model

**FIGURE 2:**
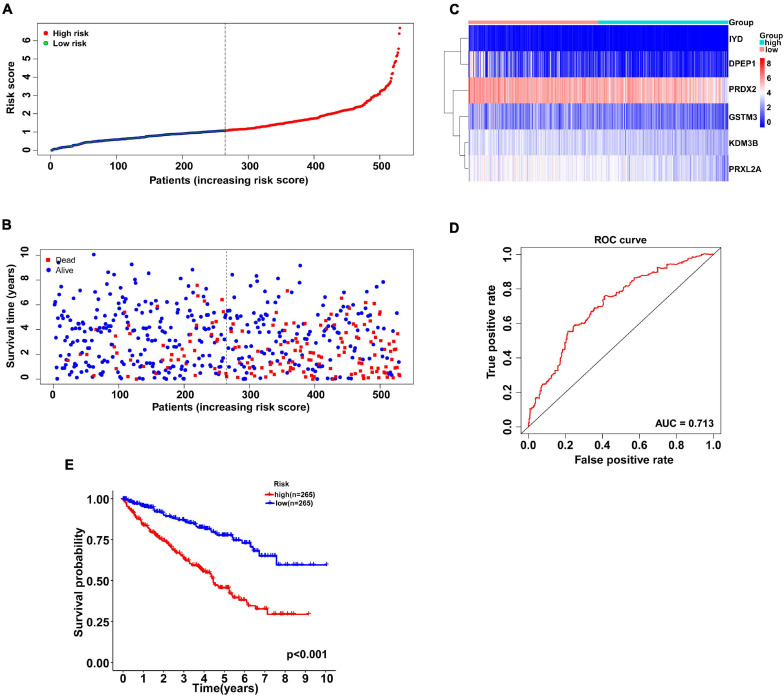
Antioxidant gene signature predicts overall survival in patients with KIRC. **(A)** The distribution of risk scores for each patient. With the median risk score as the cutoff, KIRC patients were divided into high- and low-risk subgroups. **(B)** Relationship between survival time (years) and survival status for each patient. **(C)** A heatmap of the expression profiles of six antioxidant genes in high-and low-risk subgroups. The gene expression was scaled by log_2_ (original expression of gene+1). **(D)** ROC curve of antioxidant gene signature in prognosis prediction for KIRC. The AUC was 0.713. **(E)** Kaplan-Meier curve of patients in the high- and low-risk subgroups to validate the predictive value of antioxidant gene signature. The difference between the high- and low-risk subgroups was measured by the log-rank test, with a *P*-value < 0.05. ROC, receiver operating characteristic; AUC, area under the ROC curve.

According to the multivariate Cox regression analysis, independent prognostic antioxidant genes were obtained. To evaluate the prognosis of patients, the coefficient was assigned to the formula to calculate the comprehensive risk score of six genes. Patients in high- and low-risk subgroups were grouped according to the median risk score (Figure 2A). As shown in the scatter plot, the high risk scores were mainly distributed in the poor survival interval (Figure 2B). The heatmap showed the expression chart of the six genes (Figure 2C). Additionally, the ROC curve showed that AUC was 0.713 (Figure 2D), suggesting that the prognosis evaluation of the six gene signature of patients with KIRC had good specificity and sensitivity. Similarly, the negative correlation between risk score and prognosis was affirmed by the logarithmic rank method and Kaplan-Meier survival curve (*P* < 0.001, Figure 2E).

### Relativity Between Risk Score and Clinicopathological Features

**FIGURE 3:**
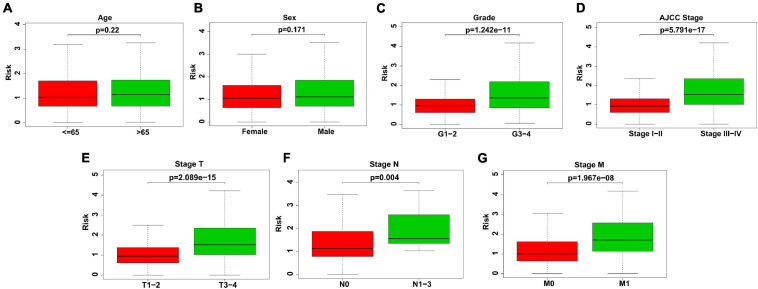
Association between risk score and clinicopathological features. **(A)** Age. No significant difference of risk scores in different age. **(B)** Sex. No significant difference of risk scores in different sex. **(C)** Grade. Risk scores were higher in KIRC patients with grade 3–4. **(D)** AJCC stage. Risk scores were higher in KIRC patients with stage III–IV (AJCC stage). **(E)** Stage T. Risk scores were higher in KIRC patients with T3–4. **(F)** Stage N. Risk scores were higher in KIRC patients with N1–3. **(G)** Stage M. Risk scores were higher in KIRC patients with M1.

We further explored whether there was a relationship between the gene signature-based risk score and clinicopathological features, consisting of age, sex, tumor grade, stage of T, N, M, and pathological stage. Our findings suggested that patients at tumor grade 3–4, stage III-IV (AJCC stage), T3-4, N1-3, and M1 stages had higher risk scores (*P* < 0.001) (Figures 3C–G). Nevertheless, the correlation of risk score and age or gender was not significant (Figures 3A,B).

### Correlation Between Risk Score and Characteristics of Immune Infiltration

**FIGURE 4:**
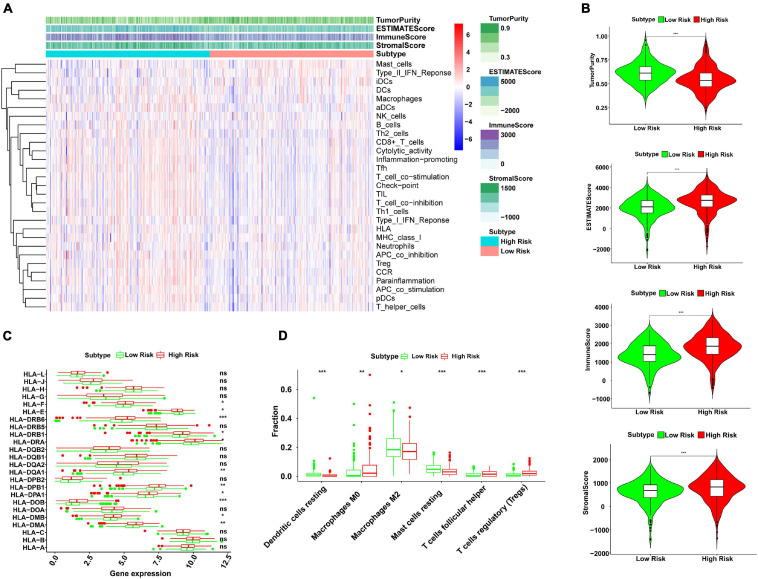
Analysis of correlation between risk score and immune infiltration profiles by ssGSEA. **(A)** The 29 immunocyte subtypes and immune-related pathways were enriched in different high- and low-risk subgroups by unsupervised consistent clustering analysis. The tumor purity, ESTIMATE score, immune score, and stromal score were calculated and are shown in the heatmap. **(B)** The violin plots showed a higher immune score, stromal score, and corresponding ESTIMATE score, and lower tumor purity in the high-risk group than the low-risk group (***P < 0.001). **(C)** The expression levels of HLA-related genes in high- and low-risk subgroups (ns, not significant, *P < 0.05, **P < 0.01, and ***P < 0.001). **(D)** The fractions of six immune cell infiltration in high- and low-risk subgroups (ns, not significant, *P < 0.05, **P < 0.01, and ***P < 0.001). ssGSEA, single sample gene set enrichment analysis; HLA, human leukocyte antigen.

SSGSEA was used to analyze 29 immune cell subtypes and immune-related pathways in each KIRC patient to study the relevance between risk score and immune infiltration profiles. The immune infiltration profiles of the risk subgroup were composed of the stromal scores, immune scores, estimate scores, and tumor purity, which were evaluated by unsupervised consistent clustering analysis (Figure 4A). Our results indicated that compared to the low-risk group, the high-risk group had higher stromal scores, immune scores, and corresponding estimate scores, as well as lower tumor purity (Figure 4B). This study also proved the significant relevance between risk score and the expression of several HLA-related genes, including HLA-F, HLA-DRB6, HLA-DRB1, HLA-DRA, HLA-DQA1, HLA-DPB1, HLA-DPA1, HLA-DMA, HLA-DMB, HLA-DOB, and HLA-E. Among the above genes, except HLA-E, the expression of all other HLA-related genes in the high-risk group was higher than that in the low-risk group (Figure 4C). The box plots intuitively showed the difference in immune cell infiltration between different risk subgroups (Figure 4D). We observed that the expressions of resting mast cells, resting dendritic cells, and M2 macrophages in the low-risk group were higher than those in the high-risk group. However, the expressions of regulatory T cells (Tregs), follicular helper T cells, and M0 macrophages in the low-risk group were lower than those in the high-risk group. The above results showed that the risk model could assess the immune condition of KIRC patients to a certain extent.

**FIGURE 5:**
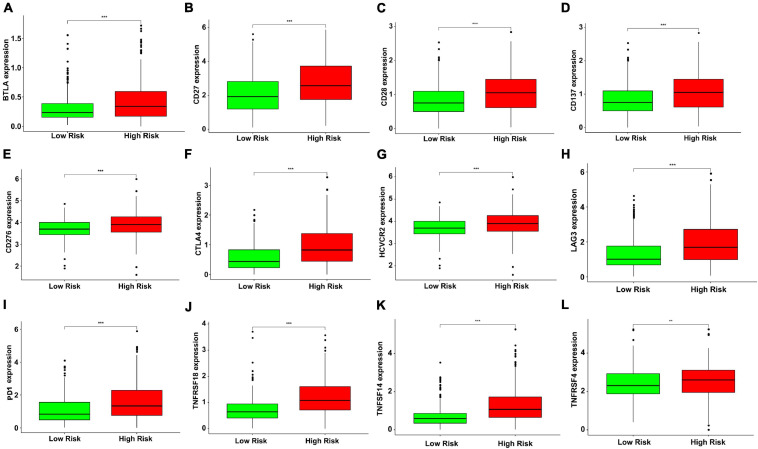
The green and red boxplots represent the expression levels of immune checkpoint genes in the low- and high-risk subgroups, respectively. The solid line represents the median expression of immune checkpoint genes. The expressions of **(A)** BTLA, **(B)** CD27, **(C)** CD28, **(D)** CD137, **(E)** CD276, **(F)** CTLA4, **(G)** HAVCR2, **(H)** LAG3, **(I)** PD1, **(J)** TNFRSF18, **(K)** TNFSF14, and **(L)** TNFRSF4 were significantly higher in the high-risk group (**P < 0.01 and ***P < 0.001). The gene expression was transformed by log_2_ (original expression of gene+1).

Besides, our study analyzed the expression levels of 12 immune checkpoint genes in different risk subgroups. The study on the relationship between risk score and gene expression of immune checkpoint provided a new idea for immunotherapy. Compared with the low-risk group, the expressions of BTLA, CD137, CD27, CD276, CD28, CTLA4, HCVCR2, LAG3, PD1, TNFRSF4, TNFRSF18, and TNFSF14 increased in the high-risk group (Figure 5), indicating that the development of potential immune checkpoint inhibitors might have an effect on high-risk patients with KIRC.

### Verification of the Predicting Power of the Six-Gene Signature

**FIGURE 6:**
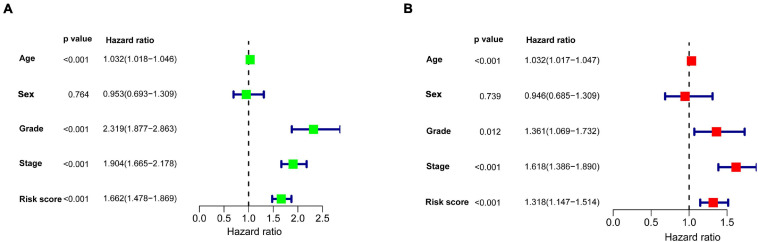
Univariate and multivariate Cox regression analyses were used to explore the impact of clinicopathological features and the gene signature on survival of patients with KIRC. **(A)** Univariate Cox regression analysis. **(B)** Multivariate Cox regression analysis. Age, grade, stage, and risk score were independent prognostic factors.

**FIGURE 7:**
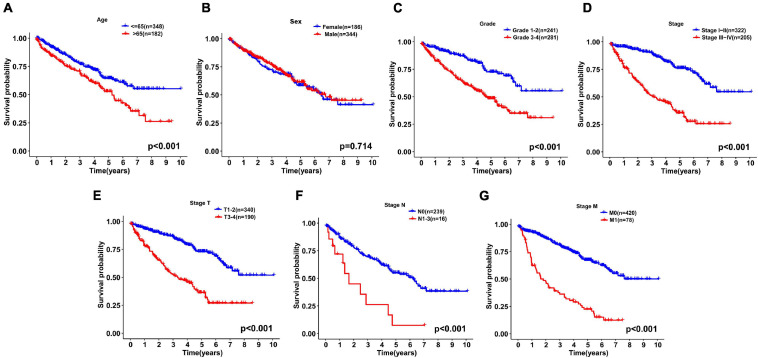
Kaplan-Meier curves for the overall survival of patients with KIRC were drawn according to the clinicopathologic features. **(A)** Age, **(B)** Sex, **(C)** Grade, **(D)** Stage, **(E)** Stage T, **(F)** Stage N, and **(G)** Stage M.

**FIGURE 8:**
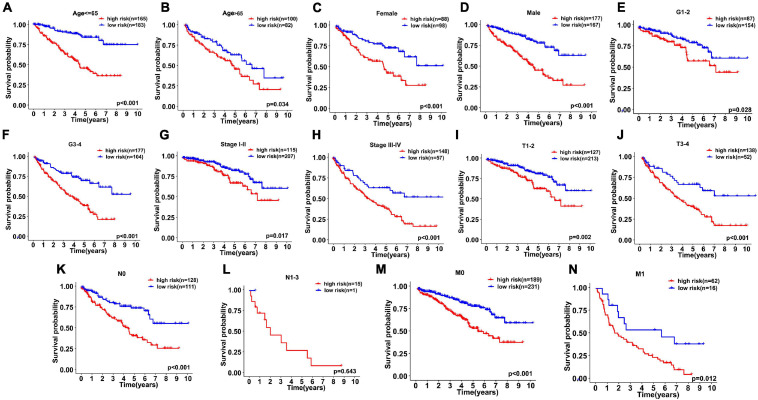
Kaplan–Meier curves for stratified survival analyses between high- and low-risk subgroups were drawn to assess the prognostic ability of gene signature. **(A)** Age ≤ 65, **(B)** Age > 65, **(C)** Female, **(D)** Male, **(E)** G1–2, **(F)** G3–4, **(G)** Stage I–II, **(H)** Stage III–IV, **(I)** T1–2, **(J)** T3–4, **(K)** N0, **(L)** N1–3, **(M)** M0, and **(N)** M1.

We continued to use univariate and multivariate analyses to investigate the predictive power of clinicopathological parameters, consisting of age, sex, grade, and stage, as well as the six-gene signature of KIRC patients for prognosis. The outcomes of the univariate analysis suggested that age [HR = 1.032, 95% confidence interval (CI): 1.018 −1.046, *P* < 0.001], grade (HR = 2.319, 95% CI: 1.877 −2.863, *P* < 0.001), stage (HR = 1.904, 95% CI: 1.665 −2.178, *P* < 0.001), and risk score (HR = 1.662, 95% CI: 1.478 −1.869, *P* < 0.001) impacted the prognosis in a significant way (Figure 6A). Likewise, multivariate analysis revealed that age (HR = 1.032, 95% CI: 1.017 −1.047, *P* < 0.001), grade (HR = 1.361, 95% CI: 1.069 −1.732, *P* = 0.012), stage (HR = 1.618, 95% CI: 1.386 −1.890, *P* < 0.001), and risk score (HR = 1.318, 95% CI: 1.147 −1.514, *P* < 0.001) were independent prognostic factors (Figure 6B). Survival curves showed that patients with age >65 years old, grade 3−4, stage III-IV, T3-4, positive lymph nodes, and distant metastasis had a poor prognosis, and sex was not significantly correlated with prognosis (Figures 7A–G). To further confirm the accuracy of the analysis in different subgroups, we conducted stratified analysis. The Kaplan-Meier curve showed (Figures 8A–N) that only in the N1-3 subgroup, risk parameters were not independent prognostic factors, and other stratifications showed that lower risk scores were associated with better outcomes.

### Construction and Validation of a Nomogram Model Combining the Antioxidant Gene Signature

**FIGURE 9:**
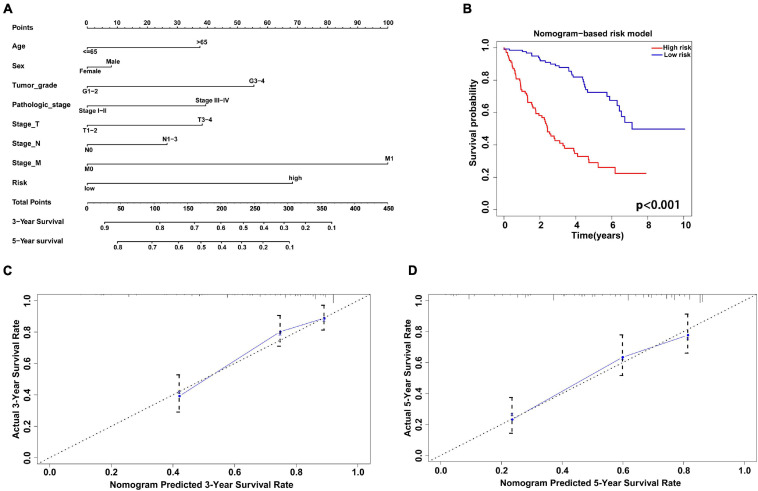
Construction and validation of nomogram model combining the antioxidant gene signature with clinicopathological factors. **(A)** The nomogram to predict 3- and 5-year survival probability of patients with KIRC. **(B)** The Kaplan-Meier curve of survival rate between two different risk groups based on the nomogram. **(C,D)** The calibration plots to estimate the 3- and 5-year predictive performance of the nomogram. Nomogram-predicted probability of survival is plotted on the *x*-axis; actual survival is plotted on the *y*-axis. The dotted line represents ideal predictive ability.

The nomogram model combining clinicopathological characteristics (age, sex, tumor grade, pathological stage, stage of T, N, M) with the risk score based on the gene signature was used to evaluate the survival probability of patients with KIRC (Figure 9A). The C-index of the survival prediction was 0.766. Additionally, the survival rate between the two risk groups based on the nomogram model was significantly different with *P* < 0.001 (Figure 9B). The calibration plots were drawn to forecast the 3- and 5-years survival rates of KIRC patients, which proved that the predicting power of the nomogram was highly concordant with the actual observation (Figures 9C,D).

## Discussion

Recently, multiple studies have shown that traditional clinicopathological characteristics, such as sex, age, stage of pathology, size of the tumor, are not high enough to predict the prognosis of patients (Zhao et al., 2019). Attention should be paid to building new models to predict the prognosis of cancer patients more effectively and accurately. With the continuous exploration of the clinical significance of molecular markers, increasing studies believe that molecular markers could predict the prognosis of tumors, and an increasing number of predictive indicators and therapeutic targets are also constantly being determined. For example, IGFLR1 has been proved to independently predict prognosis in KIRC, and the risk was higher in KIRC patients with increased IGFLR1 expression (Song et al., 2020). Based on five mRNAs and one miRNA (ANK3, GTPBP2, hsa-mir-374a, INTS8, LIMCH1, SLC16A12), Chang et al. (2018) established a good gene signature model to conduct prognosis prediction of patients with KIRC, and the results showed that the prognosis of the high-risk group was better than that of the low-risk group. In summary, although some studies have explored the molecular markers related to the prognosis of KIRC and built an available prognostic risk model, there is no study on constructing an effective prognostic model of KIRC based on antioxidant genes. The antioxidant system controls ROS production by altering metabolic and signal transduction pathways to maintain redox homeostasis in normal cells (Snezhkina, 2019). Cancer cells maintain high levels of ROS production compared to normal cells and are increasingly dependent on antioxidant defense systems (Prasad et al., 2017). It is worth noting that the role of antioxidants in tumors may change due to the development stage of tumors (Harris and DeNicola, 2020). On one hand, when the level of ROS surpasses the antioxidant defense mechanism, cells will experience oxidative stress, causing the destruction of molecules, cell structure, and function, thereby promoting the occurrence and development of tumors (Kumari et al., 2018). However, with the development of tumors to a certain extent, the level of ROS continues to rise. Cancer cells actively upregulate a variety of antioxidant systems to buffer the level of ROS, so that ROS in the tumor cells reach a new balance, which is limited to a range that is conducive to promoting tumor progression (Moloney and Cotter, 2018). Although some previous studies have proven that certain antioxidants can prevent cancer (Sakumi et al., 2003; McLoughlin and Orlicky, 2019), other studies have shown that tumors can also occur in similar situation (Müller et al., 2013; Mishra et al., 2018). These results indicate that antioxidants and ROS are closely correlated with the development of cancer, and further research is required to analyze these seemingly contradictory findings. In addition, there is an increasing number of studies on antioxidant genes, which may be involved in the signaling pathway of the antioxidant system (Wang Z. et al., 2020). The expression of these genes may be of great significance for early diagnosis, precise treatment, and prognosis evaluation of KIRC, which is worthy of further study.

In this study, the antioxidant genes were studied using bioinformatics methods, and their prognostic ability on KIRC was demonstrated. We collected antioxidant genes, analyzed the data of the KIRC and normal samples from TCGA, and discerned differentially expressed genes between them. Six independent prognostic genes (DPEP1, GSTM3, IYD, KDM3B, PRDX2, and PRXL2A) were determined through univariate and multivariate Cox regression analyses, and the six-gene signature was built to forecast the prognosis of patients with KIRC. This signature may be a more effective and targeted prognostic marker for patients with KIRC than other prognostic indicators. Meanwhile, our study found that high risk scores and late tumor stages were significantly associated with poor prognosis. Our study on the correlation between tumor infiltrating immune cells and the risk score in KIRC patients showed that the levels of Tregs, follicular helper T cells, M0 macrophages were higher in the high-risk group, while the levels of resting mast cells, resting dendritic cells, and M2 macrophages were higher in the low-risk group, which is generally similar to recent research on KIRC patients (Zhang et al., 2019; Hua et al., 2020; Fan et al., 2021). It has been suggested that overexpression of immunosuppressive molecules (e.g., CTLA4, LAG3, and PD1) and immunosuppressive cells (e.g., Tregs) are involved in tumor immune escape and contribute to tumorigenesis and progression (Liu and Cao, 2016; Saleh and Elkord, 2019). Inhibition of immune checkpoints has recently received much attention as a promising immunotherapeutic strategy in cancer (Sanmamed and Chen, 2018). Therefore, we investigated the relationship between several important immune checkpoint genes and risk scores generated from the antioxidant gene signature in KIRC. The results indicated that the expressions of gene checkpoints such as BTLA, CTLA4, CD27, CD28, LAG3, and PD1 were higher in the high-risk group in a significant way, which may help to interpret the poor outcome of KIRC in the high-risk group. And our antioxidant gene signature may also provide evidence for the appliance of immune checkpoint inhibitors in KIRC by identifying patients with positively expressed targets. Moreover, the Kaplan-Meier curve showed that the risk score was negatively related to the prognosis of patients. And a nomogram with good clinical and prognostic value was established in combination with clinical features and antioxidant gene signatures. The evidence indicates that the model based on the antioxidant gene signature is feasible in the prognosis prediction in KIRC. But our study still has some shortcomings. As shown in the stratified analysis, the risk parameters of the N1-3 subgroup could not predict the prognosis of patients with KIRC (*P* = 0.643). Insufficient samples may explain the negative results for N1-3 (*n* = 15). In addition, owing to the deficiency of recurrence data in the TCGA database, we only used OS to predict the patient’s outcomes, which may lead to the loss of some important information and affect the comprehensive application of this model.

These six genes (DPEP1, GSTM3, IYD, KDM3B, PRDX2, and PRXL2A) involved in the construction of the model have been studied by some researchers. Dipeptidase 1 (DPEP1) is situated on chromosome 16q24.3 and is a zinc-doped metalloprotease, which has a great effect on the metabolism of glutathione and leukotrienes (Nakagawa et al., 1992). Nevertheless, the molecular mechanism of cancer depends on the type and stage of cancer, and the role of DPEP1 expression in cancer is still controversial. Studies have shown that the expression of DPEP1 is reduced in invasive and *in situ* lobular carcinoma of the breast and pancreatic ductal adenocarcinoma (Green et al., 2009; Zhang et al., 2012) and is highly expressed in colorectal cancer and hepatoblastoma (Toiyama et al., 2011; Tachibana et al., 2017; Cui et al., 2019). Glutathione S-transferase mu3 (GSTM3) belongs to the phase II enzyme family of xenobiotic detoxification, which is related to the detoxification of carcinogens and metabolism of exogenous electrophiles (Li et al., 2017; Wang S. et al., 2020). Studies have shown that GSTM3 has different polymorphisms in various tumor cells and participates in the regulation of tumorigenesis, cell invasion, metastasis, chemical resistance, and oxidative stress (Hammers et al., 2017). Studies have revealed that GSTM3 obviously affects the susceptibility of individuals to cancer, such as esophageal cancer, hepatocellular carcinoma, colorectal cancer, urinary bladder cancer, breast cancer, and prostate cancer (Loktionov et al., 2001; Mitrunen et al., 2001; Jain et al., 2007; White et al., 2008; Kesarwani et al., 2009; Mitra et al., 2009). An in-depth study of the regulatory mechanism related to the abnormal expression of GSTM3 and the effect of GSTM3 in various cancers may help prevent cancers and promote targeted treatment. Iodotyrosine deiodinase (IYD) is expressed in thyroid cells and plays an essential effect in thyroid cells and thyroid hematopoietic stem cells, which catalyze the iodide cycle and promote iodine retention in thyroid follicular cells (Olker et al., 2018; Han et al., 2020). Currently, research on the IYD function is slow. Recently, Lu et al. (2020) suggested that IYD inhibits the growth of hepatocellular carcinoma cells and tumorigenesis. KDM3B is an H3K9me1/me2-specific demethylase (Xu et al., 2018). As it is located on the chromosome 5q31 region, it has been initially suspected to inhibit malignant tumors of the hematopoietic system (Xu et al., 2018). Some studies have shown that KDM3B has potential tumor-suppressive activity in myelodysplastic syndromes, acute myeloid leukemia, acute promyelocytic leukemia, and breast tumors (MacKinnon et al., 2011; Paolicchi et al., 2013; Xu et al., 2018; Wang X. et al., 2019). However, another study showed that KDM3B was located at the promoter area of the lmo2 gene and drove the occurrence of leukemia (Kim et al., 2012). Peroxiredoxin 2 (PRDX2), a member of the peroxiredoxin family, is a 2-Cys antioxidant enzyme that makes a valuable contribution to the scavenging of H2O2 and ROS, thus preventing oxidative stress in cells (De Franceschi et al., 2011). As a tumor suppressor, PRDX2 is expressed in normal melanocytes, but its expression is lost in methylated melanomas (Zhao et al., 2019). In contrast, PRDX2 overexpression is related to the development of several malignant tumors, consisting of cancers of the colon (Lu et al., 2014), cervix (Lomnytska et al., 2011), lung (Stresing et al., 2013), prostate (Ummanni et al., 2015), liver (Zhou et al., 2016), and esophagus (Feng et al., 2020). The peroxiredoxin-like 2A (PRXL2A) gene acts as an antioxidant protein to prevent cells from oxidative stress (Chen et al., 2019). It has been confirmed that overexpression of PRXL2A and lymph node metastasis is related to poor outcomes in oral squamous cell carcinoma (OSCC) patients, suggesting that downregulation of miR-125b inhibitory molecules is the basis for the upregulation of PRXL2a in OSCC (Chen et al., 2019).

In conclusion, an antioxidant gene signature was developed and verified to accurately forecast the prognosis of patients with KIRC in our study. The risk model based on antioxidant gene signature may also contribute to selecting potential patients and targets for immunotherapy in KIRC. The nomogram established by combining the gene signature and clinical factors can be used as a useful tool for prognosis evaluation in clinical management of patients with KIRC.

## Data Availability Statement

Publicly available datasets were analyzed in this study. This data can be found here: https://portal.gdc.cancer.gov.

## Author Contributions

HK and ZZ designed the study and provided methodological support. XR, LM, NW, and RZ searched the database and analyzed the data. JW, XX, and HZ prepared all the flowcharts. XR wrote the manuscript. DL, XM, and CD revised the manuscript. All authors read and approved the final manuscript before submission.

## Conflict of Interest

The authors declare that the research was conducted in the absence of any commercial or financial relationships that could be construed as a potential conflict of interest.

## Publisher’s Note

All claims expressed in this article are solely those of the authors and do not necessarily represent those of their affiliated organizations, or those of the publisher, the editors and the reviewers. Any product that may be evaluated in this article, or claim that may be made by its manufacturer, is not guaranteed or endorsed by the publisher.

## Footnotes

1
https://portal.gdc.cancer.gov/

2
https://www.gsea-msigdb.org/gsea/index.jsp
