# Supplementary material for: Antioxidant Gene Signature Impacts the Immune Infiltration and Predicts the Prognosis of Kidney Renal Clear Cell Carcinoma
Source: Front Genet. 2021 Aug 19;12:721252. doi: 10.3389/fgene.2021.721252 (PMC8416991; doi:10.3389/fgene.2021.721252)
Supplement: Supplementary file 1 [file Table_1.docx]

Supplementary Table 1 The list of four antioxidant gene sets selected from the molecular signatures database for gene set enrichment analysis.

| Gene sets | Antioxidant-related genes |
| --- | --- |
| ANTIOXIDANT_ACTIVITY | APOA4、CAT、CYGB、EPX、GPX2、GPX3、GPX4、GSR、GSTZ1、IPCEF1、MGST3、MPO、PRDX2、PRDX4、SELENOS、TXNDC2、TXNRD1、TXNRD2 |
| GO_ANTIOXIDANT_ACTIVITY | ALB、ALOX5AP、APOA4、APOE、APOM、CAT、CLIC2、CYGB、DUOX1、DUOX2、EPX、FABP1、GPX1、GPX2、GPX3、GPX4、GPX5、GPX6、GPX7、GPX8、GSR、GSTA1、GSTK1、GSTM2、GSTO1、GSTO2、GSTP1、GSTT1、GSTZ1、HBA1、HBA2、HBB、HBD、HBE1、HBG1、HBG2、HBM、HBQ1、HBZ、HP、IPCEF1、IYD、KDM3B、LOXHD1、LPO、LTC4S、MGST1、MGST2、MGST3、MPO、MT3、NQO1、NXN、PARK7、PRDX1、PRDX2、PRDX3、PRDX4、PRDX5、PRDX6、PRXL2A、PRXL2B、PRXL2C、PTGS1、PTGS2、PXDN、PXDNL、S100A9、SELENOS、SELENOT、SELENOW、SESN1、SESN2、SOD1、SOD2、SOD3、SRXN1、TP53INP1、TPO、TXN、TXNDC17、TXNDC2、TXNRD1、TXNRD2、TXNRD3、UBIAD1 |
| GO_GLUTATHIONE_CATABOLIC_PROCESS | CHAC1、CHAC2、GGT1、GGT2、GGT3P、GGT5、GGT7、GGTLC1、GGTLC2、GGTLC3 |
| GO_GLUTATHIONE_METABOLIC_PROCESS | ALDH5A1、CHAC1、CHAC2、CNDP2、CTNS、DPEP1 、ETHE1、G6PD、GCLC、GCLM、GGCT、GGT1、GGT2、GGT3P、GGT5、GGT6、GGT7、GGTLC1、GGTLC2、GGTLC3、GLO1、GLRX2、GPX1、GPX4、GSR、GSS、GSTA1、GSTA2、GSTA3、GSTA4、GSTA5、GSTK1、GSTM1、GSTM2、GSTM3、GSTM4、GSTM5、 GSTP1、GSTT1、GSTT2、GSTT2B、GSTZ1、HAGH、HPGDS、IDH1、MGST1、MGST2、MMACHC、NAT8、 NFE2L1、NFE2L2、OPLAH、PARK7、PTGES、SLC7A11、SOD1 |
