# Supplementary material for: Antioxidant Gene Signature Impacts the Immune Infiltration and Predicts the Prognosis of Kidney Renal Clear Cell Carcinoma
Source: Front Genet. 2021 Aug 19;12:721252. doi: 10.3389/fgene.2021.721252 (PMC8416991; doi:10.3389/fgene.2021.721252)
Supplement: Supplementary file 2 [file Table_2.docx]

Supplementary Table 2 Differentially expressed antioxidant genes between KIRC and normal samples.

|  | Differentially expressed genes |
| --- | --- |
| Up-regulation | SELENOS、MGST2、GCLM、GSTO1、SELENOW、GPX4、GPX7、MGST1、GSTM1、HPGDS、GGT1、CNDP2、PRXL2C、GPX1、GPX8、LOXHD1、HBG2、CLIC2、TP53INP1、S100A9、TXNDC2、LTC4S、SOD2、ALOX5AP、PRDX4、HBQ1、GGT2、PXDN、SLC7A11、GGT3P、HBM、TPO、CYGB、MT3、HP |
| Down-regulation | GGT6、FABP1、DPEP1、IYD、GSTM3、CHAC1、GSTO2、GPX3、SOD3、EPX、ALB、CAT、GSTM5、GPX2、PTGS2、APOM、ALDH5A1、GGTLC1、IPCEF1、DUOX2、PTGES、CHAC2、PRDX3、MMACHC、HAGH、IDH1、GGTLC2、GSR、GSTZ1、TXNRD2、GSTP1、SESN2、PRDX1、OPLAH、PRXL2A、GLO1、NFE2L2、PRDX2、CTNS、SOD1、GGT5、NQO1、TXNRD3、PRDX6、GSTM4、GGCT、NFE2L1、UBIAD1、GSTT2B、SELENOT、GGT7、PRDX5、LPO、KDM3B、PARK7、THE1、HBD |
