# Supplementary material for: Antioxidant Gene Signature Impacts the Immune Infiltration and Predicts the Prognosis of Kidney Renal Clear Cell Carcinoma
Source: Front Genet. 2021 Aug 19;12:721252. doi: 10.3389/fgene.2021.721252 (PMC8416991; doi:10.3389/fgene.2021.721252)
Supplement: Supplementary file 3 [file Table_3.doc]

Supplementary Table 3 The fold-change and p-value of differentially expressed antioxidant genes between KIRC and normal samples.

| **Differentially expressed genes** | **Log_2_（fold change）** | **P-value** |
| --- | --- | --- |
| GGT6 | -4.928027927 | 3.39E-40 |
| FABP1 | -4.551502641 | 3.06E-18 |
| DPEP1 | -4.287767638 | 3.23E-23 |
| IYD | -4.213997514 | 3.37E-29 |
| GSTM3 | -3.331205991 | 4.21E-41 |
| CHAC1 | -2.023703384 | 8.91E-28 |
| GSTO2 | -1.808071815 | 1.97E-33 |
| GPX3 | -1.560492479 | 5.82E-05 |
| SOD3 | -1.554854117 | 5.04E-32 |
| EPX | -1.429241611 | 4.15E-17 |
| ALB | -1.34855351 | 5.03E-16 |
| CAT | -1.274063024 | 2.08E-33 |
| GSTM5 | -1.261563923 | 2.92E-17 |
| GPX2 | -1.249300635 | 4.76E-28 |
| PTGS2 | -1.192518566 | 2.04E-14 |
| APOM | -1.186972901 | 0.00079868 |
| ALDH5A1 | -1.036493886 | 4.30E-30 |
| GGTLC1 | -0.968986511 | 5.98E-11 |
| IPCEF1 | -0.965574756 | 3.23E-22 |
| DUOX2 | -0.954642296 | 1.98E-23 |
| PTGES | -0.909013822 | 1.70E-16 |
| CHAC2 | -0.902678056 | 7.21E-29 |
| PRDX3 | -0.885479327 | 6.52E-35 |
| MMACHC | -0.801896918 | 3.13E-24 |
| HAGH | -0.694510999 | 1.16E-13 |
| IDH1 | -0.616520138 | 1.98E-07 |
| GGTLC2 | -0.616153463 | 1.77E-07 |
| GSR | -0.59588715 | 9.27E-26 |
| GSTZ1 | -0.579459254 | 8.77E-23 |
| TXNRD2 | -0.552492446 | 7.00E-23 |
| GSTP1 | -0.549711309 | 5.58E-08 |
| SESN2 | -0.549684678 | 1.40E-08 |
| PRDX1 | -0.535132342 | 6.60E-24 |
| OPLAH | -0.527816377 | 1.18E-13 |
| PRXL2A | -0.527517534 | 4.01E-14 |
| GLO1 | -0.502433855 | 1.44E-21 |
| NFE2L2 | -0.484936507 | 9.33E-19 |
| PRDX2 | -0.474559299 | 1.94E-17 |
| CTNS | -0.454366608 | 5.66E-20 |
| SOD1 | -0.451491491 | 5.20E-21 |
| GGT5 | -0.44693755 | 8.33E-06 |
| NQO1 | -0.424650323 | 1.22E-12 |
| TXNRD3 | -0.412066535 | 2.03E-16 |
| PRDX6 | -0.379058866 | 1.10E-21 |
| GSTM4 | -0.351675327 | 1.90E-13 |
| GGCT | -0.331647306 | 6.78E-16 |
| NFE2L1 | -0.302313354 | 3.60E-14 |
| UBIAD1 | -0.299274195 | 7.84E-11 |
| GSTT2B | -0.247431307 | 0.040470494 |
| SELENOT | -0.231588179 | 2.63E-09 |
| GGT7 | -0.214226243 | 2.98E-05 |
| PRDX5 | -0.165779122 | 1.30E-06 |
| LPO | -0.120606379 | 1.47E-07 |
| KDM3B | -0.109251976 | 0.010130007 |
| PARK7 | -0.096563502 | 0.000520798 |
| ETHE1 | -0.071260814 | 0.009944392 |
| HBD | -0.004904718 | 1.13E-17 |
| SELENOS | 0.173465761 | 0.000409634 |
| MGST2 | 0.220484371 | 0.001203284 |
| GCLM | 0.255854865 | 0.001559029 |
| GSTO1 | 0.397153259 | 5.09E-07 |
| SELENOW | 0.4963785 | 5.80E-13 |
| GPX4 | 0.501080478 | 1.17E-07 |
| GPX7 | 0.54584174 | 1.73E-07 |
| MGST1 | 0.578077119 | 2.08E-05 |
| GSTM1 | 0.600655822 | 0.023863884 |
| HPGDS | 0.635409336 | 5.63E-08 |
| GGT1 | 0.650026079 | 1.31E-06 |
| CNDP2 | 0.709683167 | 9.21E-13 |
| PRXL2C | 0.776544205 | 3.75E-25 |
| GPX1 | 0.786001735 | 1.22E-15 |
| GPX8 | 0.83453695 | 1.81E-14 |
| LOXHD1 | 0.866418061 | 0.036034507 |
| HBG2 | 0.885812042 | 5.17E-06 |
| CLIC2 | 0.896027483 | 5.97E-19 |
| TP53INP1 | 0.978059382 | 2.17E-26 |
| S100A9 | 1.070042126 | 2.79E-10 |
| TXNDC2 | 1.108973653 | 8.16E-08 |
| LTC4S | 1.153698831 | 1.72E-07 |
| SOD2 | 1.188816985 | 4.61E-15 |
| ALOX5AP | 1.193537197 | 2.67E-15 |
| PRDX4 | 1.407871578 | 2.61E-36 |
| HBQ1 | 1.461760626 | 0.028961182 |
| GGT2 | 1.585072368 | 0.00356821 |
| PXDN | 1.671774618 | 7.10E-25 |
| SLC7A11 | 1.759740922 | 1.52E-25 |
| GGT3P | 1.810381396 | 5.51E-05 |
| HBM | 1.875974519 | 0.011302419 |
| TPO | 1.979732142 | 9.09E-15 |
| CYGB | 2.020240607 | 2.69E-29 |
| MT3 | 3.276867852 | 1.05E-05 |
| HP | 7.155818989 | 0.000889275 |
