# Supplementary figures and images for: Antioxidant Gene Signature Impacts the Immune Infiltration and Predicts the Prognosis of Kidney Renal Clear Cell Carcinoma
Source: Front Genet. 2021 Aug 19;12:721252. doi: 10.3389/fgene.2021.721252 (PMC8416991; doi:10.3389/fgene.2021.721252)

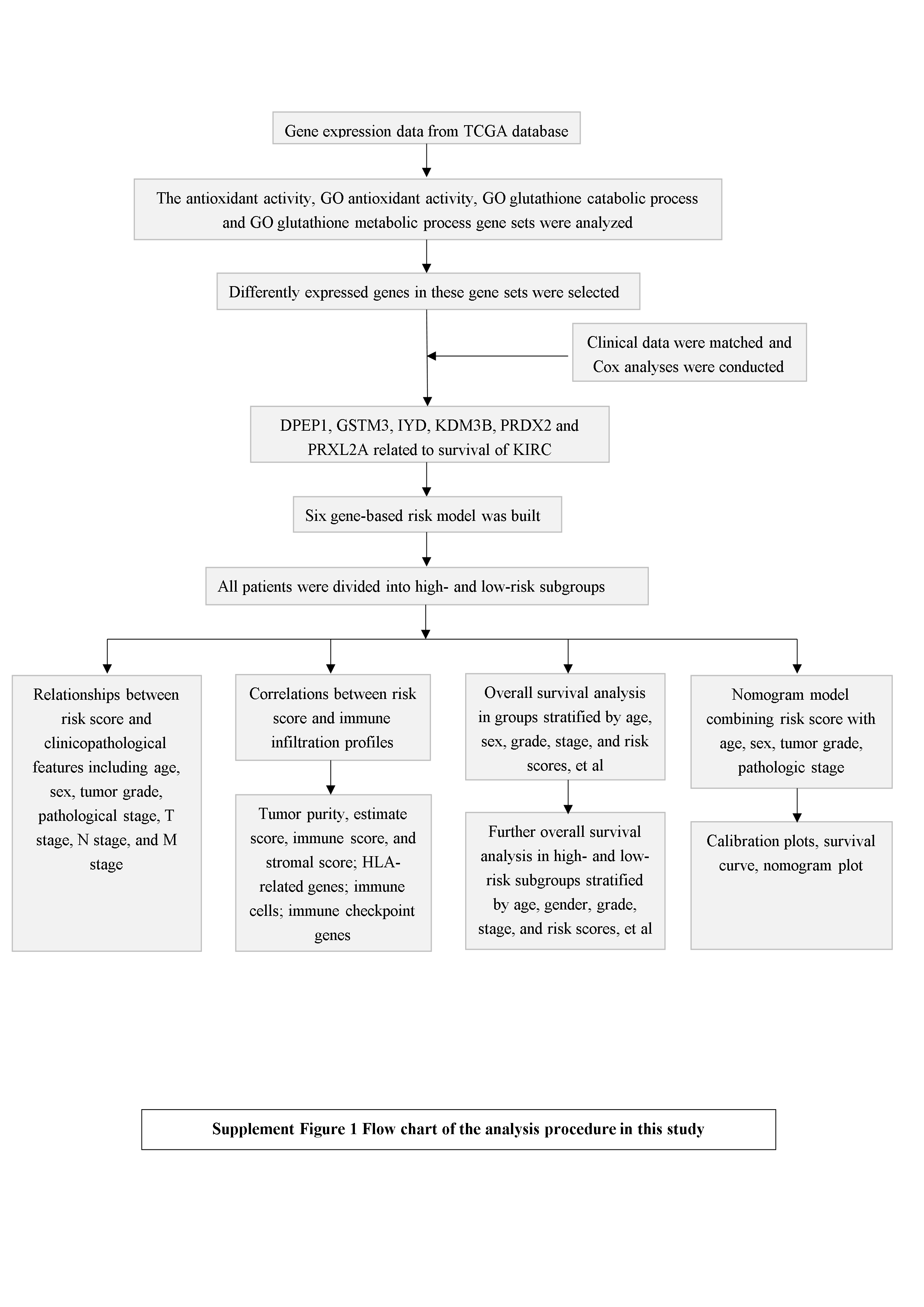

Supplement: Supplementary file 4 [file Image_1.TIF]
